# Prevalence and Associated Mortality of Infections by Multidrug-Resistant Organisms in Pediatric Intensive Care Units in Argentina (PREV-AR-P)

**DOI:** 10.3390/antibiotics14050493

**Published:** 2025-05-11

**Authors:** Wanda Cornistein, Carina Balasini, Yanina Nuccetelli, Viviana M. Rodriguez, Norma Cudmani, Maria Virginia Roca, Graciela Sadino, Martín Brizuela, Analía Fernández, Soledad González, Damián Águila, Alejandra Macchi, Maria Inés Staneloni, Elisa Estenssoro

**Affiliations:** 1 Hospital Universitario Austral, Buenos Aires 1629, Argentina; wcornist@cas.austral.edu.ar; 2 Argentinian Society of Infectious Diseases (SADI), Buenos Aires 1414, Argentina; yanina.nuccetelli@hospitalespanol.org.ar (Y.N.); viviana-rodriguez@buenosaires.gob.ar (V.M.R.); dir_investigacion@msptucuman.gov.ar (N.C.); infectologia@asocespa.com (M.V.R.); gracielasadino@curf.ucc.edu.ar (G.S.); segonzalez@ms.gba.gov.ar (S.G.); damian.aguila@unr.edu.ar (D.Á.); amacchi@sanatoriolaslomas.com (A.M.); maria.staneloni@hospitalitaliano.org.ar (M.I.S.); 3 Argentinian Society for Critical Care (SATI), Buenos Aires 1414, Argentina; sc.balasini@buenosaires.gob.ar (C.B.); analiafernandez@buenosaires.gob.ar (A.F.); eestenssoro@med.unlp.edu.ar (E.E.); 4 Hospital Pirovano, Buenos Aires 1430, Argentina; 5 Hospital Interzonal de Agudos San Martín de La Plata, Buenos Aires 1900, Argentina; 6 Hospital Tornú, Buenos Aires 1427, Argentina; 7 Departamento de Control de Infecciones y Gestión de Antimicrobianos, Ministerio de Salud Pública, Tucumán 4000, Argentina; 8 Hospital Zonal Alvear, Comodoro Rivadavia 9001, Argentina; 9 Clinica Universitaria Reina Fabiola, Córdoba 5000, Argentina; 10 Hospital General de Agudos Dr. Juan A. Fernández, Buenos Aires 1425, Argentina; 11 Hospital Durand, Buenos Aires 1405, Argentina; 12 Ministerio de Salud de la Provincia de Buenos Aires, Buenos Aires 1900, Argentina; 13 Hospital Provincial del Centenario, Rosario 2000, Argentina; 14 Sanatorio Las Lomas, Buenos Aires 1609, Argentina; 15 Hospital Italiano de Buenos Aires, Buenos Aires 1900, Argentina

**Keywords:** Argentina, pediatric intensive care units, carbapenem-resistant *Enterobacterales*, colonization, infection, prevalence, multidrug resistance

## Abstract

**Background/Objectives:** Data on multidrug-resistant organism (MDRO) infections in children are scarce, especially in resource-limited regions. This study aimed to estimate the prevalence of MDRO infections in pediatric intensive care units (PICUs) and characterize their epidemiologic and clinical features. **Methods:** A national, multicenter, point-prevalence study was conducted in 50 PICUs in Argentina over 24 h between 24 and 28 November 2023. The primary study outcome was the prevalence of ICU infections caused by MDROs. Secondary outcomes included the prevalence of carbapenemase-producing *Enterobacterales* (CPE) colonization, ICU mortality, and ICU length of stay (LOS_ICU_). **Results:** 304 patients were included. The overall prevalence of infection was 45.1% (137/304); of these, 50.3% (69/137) were hospital-acquired. Among the 137 patients with reported infections, 49.6% (*n* = 68) were classified as definite (microbiologically confirmed) and 50.4% (*n* = 69) as probable (no confirmatory microbiology). Among definite infections, 20.6% (*n* = 14) were due to MDROs. The overall prevalence of MDRO infections was 4.6% (14/304). Extended-spectrum β-lactamase (ESBL)-producing organisms were the most commonly identified microorganisms (42.9%), followed by CPE (28.6%). Ventilator-associated pneumonia (VAP) was the most frequent location of MDRO infections. The prevalence of CPE colonization was 13.2%. Mortality was low (5.3%) and similar in patients with MDRO and non-MDRO infections. LOS_ICU_ was longer in patients with MDRO infections compared to patients with non-MDRO infections (81 [22–150] vs. 25 [12–27] days, respectively, *p* = 0.0007). **Conclusions:** Among 304 PICU patients, the prevalence of MDRO infections and colonization was relatively low. MDRO infections were not associated with increased mortality but were associated with longer ICU stays, compared to patients with non-MDRO infections.

## 1. Introduction

Multidrug-resistant organism (MDRO) infections represent a substantial challenge to healthcare systems globally, contributing to increased morbidity, mortality, extended hospitalizations, and increased healthcare costs, particularly in resource-limited settings [1]. In 2019, the World Health Organization (WHO) recognized antimicrobial resistance (AMR) as a global health threat with an estimated 5 million deaths associated with MDRO infections worldwide [2,3,4]. In the Americas, a recent study estimated 141,000 attributable deaths (95% CI: 99,900–196,000) to MDRO infections [4]. Lower respiratory and thoracic infections posed the highest AMR-related mortality risk, followed by bloodstream and peritoneal/intra-abdominal infections. The primary pathogens implicated in AMR-related deaths included *Staphylococcus aureus*, *Escherichia coli*, *Klebsiella pneumoniae*, *Streptococcus pneumoniae*, *Pseudomonas aeruginosa*, and *Acinetobacter baumannii* [2,3,4].

Patients admitted to the Intensive Care Unit (ICU), where invasive devices are common, are particularly vulnerable. Additional risk factors exist in pediatric populations, such as prematurity, which has been cited as significant for both colonization and infection with multidrug-resistant organisms (MDROs) [5,6,7,8,9,10,11,12]. However, data on antimicrobial resistance (AMR) in children are scarce, especially in resource-limited regions. According to some studies from Latin America, the mortality rate for MDRO infections in children ranges from 14% to 30% [5,12].

The limited information regarding the prevalence, resistance patterns, and clinical presentations of multidrug-resistant organisms (MDROs) in pediatric intensive care units (PICUs) in resource-limited settings represents a significant knowledge gap. Addressing this gap is the first step toward guiding preventive strategies and improving treatment guidelines. An additional challenge facing the region is the scarcity of new and effective antibiotics available for treating MDROs in children, often resulting in off-label use [13,14,15].

With the objective of addressing this knowledge scarcity regarding the current epidemiology of MDRO infections and colonization in PICUs in Argentina, as well as the associated mortality, the Argentine Society of Infectious Diseases (SADI) and the Argentine Society of Intensive Care Medicine (SATI) conducted this multicenter point-prevalence study. Based on previous research indicating prevalence rates of MDROs exceed 30% in high-risk adult ICU settings, we hypothesized that PICUs might see similarly high rates, requiring targeted surveillance and intervention strategies [1,2,14,16].

## 2. Results

### 2.1. Characteristics of Participating Centers

This study included 50 pediatric ICUs across Argentina. Hospitals were mainly public (73.6%, *n* = 37) and general (66%, *n* = 33). A total of 96.3% (*n* = 48) had an infection control and prevention committee, and 47.2% (*n* = 24) had an antimicrobial stewardship program. Other characteristics are shown in Table 1.

### 2.2. Characteristics of the Entire Population

We included 304 patients, with a median of 3 [2–8] patients per center. The median patient age was 27 [5–88] months, and most were male (55.2%, *n* = 168). In 32.5% (*n* = 99) of the included patients, a comorbidity was detected, the most frequent being respiratory disease (20.4%, *n* = 62), prematurity (17.1%, *n* = 52), and bronchopulmonary dysplasia (12.8%, *n* = 39). The main cause of hospital admission was a medical condition in 76.6% (*n* = 232).

The overall prevalence of infection was 45.1% (*n* = 137), with 50.4% (*n* = 69) being hospital-acquired. A total of 32.9% (*n* = 45) and 8.5% (*n* = 12) presented with sepsis and septic shock, respectively. Comparisons between patients with (*n* = 137) and without infection (*n* = 167) are shown in Table 2.

### 2.3. Prevalence and Characteristics of Patients with MDRO Infections

**Figure 1:**
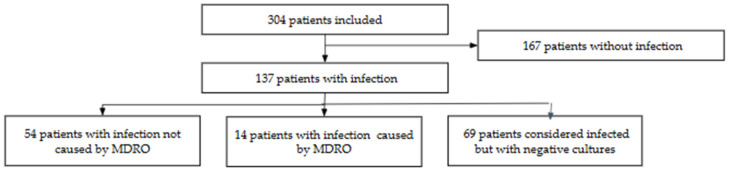
Patient enrollment by infection status and type (*n* = 304).

Among the 137 patients with a reported infection, 49.6% (*n* = 68) were classified as definite infection (microbiologically confirmed), and 50.4% (*n* = 69) as having a probable infection (no confirmatory microbiology); see Figure 1. Among definite infections, 63.2% (*n* = 43) were due to Gram-negative pathogens, and 36.8% (*n* = 25) were due to Gram-positive pathogens. A total of 20.6% (*n* = 14) were classified as MDRO and 79.4% (*n* = 54) as non-MDRO.

The prevalence of MDRO infections was 4.6% (14/304). The clinical and epidemiologic characteristics of patients with MDRO infections (*n* = 14) and non-MDRO infections (*n* = 54) are shown in Table 3.

### 2.4. Prevalence of CPE Colonization

The overall prevalence of CPE colonization was 13.2% (*n* = 40). Sixty-six percent of patients with an MDRO infection became colonized with CPE during their hospital stay, in comparison to patients without an MDRO infection, where only 20.5% of such patients became colonized during their stay (*p* = 0.002), see Table 3.

### 2.5. Sites and Microorganisms Isolated

The location of the 14 MDRO infections was as follows: ventilator-associated pneumonia (42.9%, *n* = 6), catheter-related bloodstream infection (21.4%, *n* = 3), community-acquired pneumonia (14.3%, *n* = 2), catheter-related UTI, intra-abdominal infection, and surgical site infection (7.1%, *n* = 1 each). Among non-MDRO infections (*n* = 54), the location distribution was as follows: ventilator-associated pneumonia and community-acquired pneumonia (23.6%, *n* = 17 each), catheter-related bloodstream infection (11.1%, *n* = 8), catheter-related UTI (8.3%, *n* = 6), and others (29.2%, *n* = 21).

**Figure 2:**
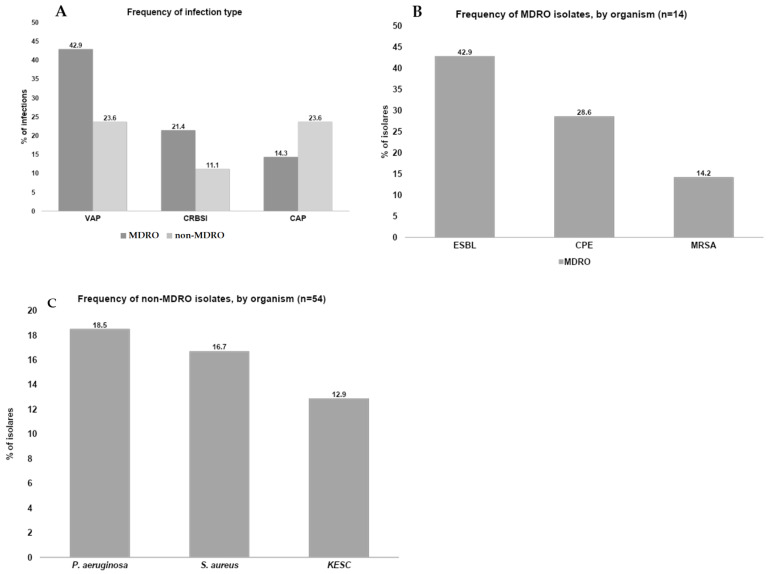
Characteristics of MDRO and non-MDRO infections. (**A**) VAP, ventilator-associated pneumonia; CRBSI, catheter-related bloodstream infection; CAP, community-acquired pneumonia. (**B**) ESBL, extended-spectrum β-lactamase-producing microorganisms; CPE, carbapenemase-producing *Enterobacterales*; MRSA, methicillin-resistant *Staphylococcus aureus.* (**C**) KESC group, including *Klebsiella*, *Enterobacter*, *Serratia*, and *Citrobacter*; *S*. *aureus*, *Staphylococcus aureus*; *P. aeruginosa*, *Pseudomonas aeruginosa*.

The most common MDRO phenotypes were ESBL (42.6%, *n* = 6), CPE (28.5%, *n* = 4), and MRSA (14.2%, *n* = 2). No MDRO co-infections were found; each of the 14 patients had a single MDRO infection. The mechanisms of CPE resistance identified were OXA (50%, *n* = 2), MBL (25%, *n* = 1), and KPC (25%, *n* = 1). The most frequent bacteria identified in the non-MDRO group were *P. aeruginosa* (18.5%, *n* = 10), *S. aureus* (16.7%, *n* = 9), and the KESC group (12.9%, *n* = 7). There were co-infections in some patients with an average of 1.3 infections per patient (72/54) **(Figure 2** and Table 4).

Patients with and without MDRO infections received targeted and adequate antibiotic treatments with similar distribution. Patients with MDRO infections received new and reserved antibiotics per WHO classification in 21.4% and 28.6% of the cases, respectively. Table 3.

### 2.6. ICU Outcomes

The overall mortality rate was 5.3% [3–8.4%] (*n* = 16/304). Mortality rate, ICU, and hospital stay are shown in Table 2 and Table 3. The determination of independent predictors of ICU mortality and MDRO infections was not performed by logistic regression analysis due to the low number of events (MDRO infections and ICU and deaths).

## 3. Discussion

The most relevant finding of the PREVAR-P study, carried out in pediatric ICUs, was the high total infection rate (45.1%), with half of the infections (22.6%) being hospital-acquired. The prevalence of MDRO infections was 4.6%, substantially lower than the 15.1% reported in the adult PREVAR study [16]. MDROs made up 20.6% of microbiologically confirmed infections, which was also lower than the 45.6% reported in the adult study [16]. ICU LoS was significantly longer in patients with MDRO infections compared to patients with non-MDRO.

High rates of hospital-acquired infections are usually the result of gaps in infection control programs, particularly among vulnerable populations and in low-resource settings. Although most of the participating ICUs had an infection control committee, assessing the functionality of such committees was not an objective of this study. In adult studies, the prevalence of healthcare-associated infections has been estimated to range from 5.7% to 19.1% in low and middle-income countries, and 5.7 to 7.5% in high-income countries [17]. Data from the pediatric population are scarce; to our knowledge, this is the first large-scale point-prevalence study in Latin America of MDRO infections, CPE colonization, and mortality carried out exclusively in pediatric ICUs. Over the last decade, six point-prevalence studies focusing on healthcare-associated infections in pediatrics have been published, primarily in European countries [18,19,20,21,22,23]. They report an 11.9 to 37% prevalence, similar to the 22.6% we found in our population. The European Centre for Disease Prevention and Control (ECDC) surveillance data from 2023 reported a slight decrease in healthcare-associated infections from 15.5% to 13%, when compared to their previous point-prevalence survey in 2015. Both analyses included more than 1000 hospitals with approximately 17,000 patients [21,23].

Among the most frequent predisposing factors for infection, we found underlying conditions such as prematurity, bronchopulmonary dysplasia, congenital heart disease, and oncologic/hematologic disease, which are similar to other studies in Portugal and Italy [24,25]. Patients with infections had higher SOFA scores at admission and on the study day, and had more frequent medical admissions (vs. surgical). These findings align with other studies and highlight the significant association between severity scores and infection [26,27,28].

The prevalence of MDRO infections was 4.3%. The ECDC surveillance report covering data from 2022 to 2023 included adult and pediatric populations and described a prevalence of CPE of 9.3%, with rates as high as 40.8% and 42.9% in Greece and Romania, respectively. In this report, the resistance to third-generation cephalosporins was 34.7% among *Enterobacterales* [23]. Studies with different methodologic designs reported rates of MDRO infections in the pediatric population ranging from 9.9% to 42% across different countries and regions [29,30,31]. In pediatric ICUs from Spain, the rate of MDRO among Gram-negative bacteria was 9.9%, with a notable increase in multidrug-resistant *Enterobacterales* over time [29]. A point-prevalence study of MDRO infections in children in Vietnam found that 35% of infections were caused by ESBL-producers and 40% by CPE [20]. In China, the Infectious Disease Surveillance of Pediatrics (which included 11 tertiary care children’s hospitals) reported high rates of MDRO, with a rate of 19.7% for carbapenem-resistant *Klebsiella pneumoniae*, 46.4% for carbapenem-resistant *Acinetobacter baumannii* (CRAB), and 35.0% for methicillin-resistant *Staphylococcus aureus* [30]. In a recent cross-sectional study in Saudi Arabia, MDROs were isolated in 42% of patients, including many patients admitted to the NICU and PICU [31].

The risk factors for the development of an infection identified in this study were the presence of pre-existing diseases and the severity of acute illness. The risk factors for MDRO infections included longer duration of ICU and hospital stay prior to the study day, prior MDRO colonization, and acquisition of MDRO colonization during the incident hospital admission. This underscores the relevance of prolonged exposure to the healthcare system to the development of MDRO infection. Inadequate infection prevention and control measures, along with a lack of antimicrobial stewardship programs, further increase the risk for the development of MDRO infections [32,33,34,35].

The prevalence of CPE colonization in this study was 13.2%, which is similar to colonization rates reported in the literature, which range from 4% to 39% [12,32,36,37,38]. CPE colonization acquired during the present hospitalization was higher in patients with MDRO infections, in comparison to those with non-MDRO infections. Most studies report a strong association between prior colonization with CPE and subsequent MDRO infections, highlighting the importance of individually tailored empiric treatment regimens in patients colonized with CPE who present with infection. Risk factors for colonization in hospitalized pediatric patients usually include previous antibiotic administration, chronic and complex comorbidities, and the presence of invasive medical devices. Effective management of colonization risk in this population involves strategies like targeted screening followed by contact precautions, and robust antibiotic stewardship activities [12,32,36,37,38].

Regarding clinical presentation, the prevalence of sepsis and septic shock in the entire study population (41.7%) was similar to the findings in a study from Spain (37.4%), but higher than reported from Brazil (25.0%), where only severe sepsis was considered, and Portugal (30%) [24,27,39]. There were no differences in the prevalence of sepsis and septic shock between patients with MDRO and non-MDRO infections.

The mortality rate was low (5.3%), and similar between patients with MDRO and non-MDRO infections. In other studies, the mortality associated with MDRO infections has ranged between 11 and 19%, depending on the microorganisms isolated, mechanisms of resistance present, and sites of infection [19,24,27,31,32,37,40,41,42]. The low mortality found in our study may be explained by the low number of MDRO infections and the likely high rate of well-tailored empirical treatment. Usually, empiric antibiotic treatments used in pediatric ICUs include coverage for ESBL, PAE, and MRSA, which were the most common MDROs in this study. Additionally, the low mortality might reflect changes in global trends such as decreases in the worldwide estimates of AMR-associated mortality, which has decreased by 63.3%, from 1990 to 2021, in children <5 years (the median age of our patients was 27 months) [43]. However, in children ≥5 years, studies have shown that AMR-associated mortality increased, in absolute numbers, from 2.49 million in 1990 to 3.87 million in 2021 [43].

MDRO infections were associated with significantly longer ICU and hospital stays. These findings align with previous studies describing an extended pediatric ICU length of stay in patients with MDRO infections, without significant differences in mortality [21,32].

Half of the infected patients had a definite infection; most studies found similar rates (44–58%) [19,21]. The MDRO isolates were also similar to previous publications in that there was a predominance of Gram-negative bacilli, particularly *Enterobacterales*, followed by non-fermenting microorganisms and MRSA. The resistance phenotypes and mechanisms identified, such as ESBL producers, CPE, and MRSA, are consistent with global patterns, yet there are some regional genetic variations regarding carbapenemase mechanisms [16,24,31,37,41,44,45,46].

Ventilator-associated pneumonia and catheter-associated bloodstream infections emerged as the most common type of MDRO infections in our study, corroborating findings of previous research [19,21,24,31,32,37,45].

As mentioned previously, the prevalence of MDRO infections in the PREVAR-P study was lower than the prevalence reported in the PREVAR study in adults; the CPE colonization rate was also lower by half [16]. MDROs accounted for 20.6% of microbiologically confirmed infections in PREVAR-P, significantly lower than the 45.6% reported in PREVAR [16]. Several factors might account for these differences between the pediatric and adult populations. As the microbiome composition changes with aging, the expression of antibiotic-resistance genes might increase, especially in the critically ill [47]. In addition, adults experience higher cumulative antibiotic exposure over time due to more interactions with the healthcare system and the treatment of chronic diseases, which can select resistant microorganism strains [48]. Finally, adults are more likely to receive broad-spectrum antibiotics due to differences in antibiotic prescribing practices [49].

This study has several limitations. First, the point-prevalence design does not allow us to establish causation in the associations highlighted in this paper. In addition, hospital-acquired infections might be overestimated given that they are associated with longer ICU stays, increasing the likelihood of being sampled. Second, hospital participation was voluntary; thus, selection bias might be possible. Third, the number of infections with MDROs was low; therefore, we could not identify independent predictors (risk factors) via logistic regression. A similar caveat might be present regarding the identification of independent predictors of mortality. Fourth, the total number of MDRO infections may be an underestimate, since 50.4% of patients had unconfirmed infections. However, this also occurred in other studies. Finally, we did not have detailed information on patient antibiotic treatments and were thus unable to evaluate how access to new drugs might have contributed to our results. Despite these drawbacks, the large number of pediatric ICUs—both public and private—is a strength of this study.

## 4. Patients and Methods

The PREVAR-P (PREValence of infection with MDRO in Argentina in the Pediatric population) was a national, multicenter, point-prevalence study performed in 50 pediatric ICUs in Argentina. The study was also carried out simultaneously in an adult ICU population [16]. Data at each site were collected over a 24 h period, with all data collection conducted between 24 and 28 November 2023. Hospitals were registered to participate in the study utilizing a secure website, where local investigators recorded hospital characteristics in an electronic form. Definitions and procedures are shown in the Manual of Operations (Appendix A) [16].

Each local institutional review board reviewed and provided ethical approval, establishing the requirement for informed consent.

All pediatric patients, defined as those aged between 1 month and 18 years who were admitted to the participating ICUs during the 24 h study period (beginning at 8.00 a.m.), were included. Patients were followed up for ICU mortality. There were no exclusion criteria. Patient data were entered into an electronic case report form (CRF) using the Research Electronic Data Capture (REDCap) database. A centralized data center managed the database and monitored data quality to minimize missing data, in collaboration with local researchers. Patient data were anonymized by assigning a study ID.

The CRF included baseline demographic characteristics, such as the date of hospital and ICU admission, age, sex, comorbid conditions, and admission Sequential Organ Failure Assessment (SOFA) scores [50]. SOFA score was also calculated and recorded on the day of the study. Data on risk factors for MDRO infection during the prior 6 months was collected, including prior hospital admission and history of colonization with carbapenemase-producing *Enterobacterales* (CPE). Regarding prior MDRO colonization, only colonization data on CPE were included, as it is the most common type of MDRO screening performed in Argentine ICUs.

On the day of the study, the treating physician recorded the type of admission as medical or surgical (elective or emergency) and the presence of sepsis or septic shock [51,52]. Gastrointestinal CPE colonization data were recorded from rectal swabs taken during the incident hospitalization.

Infections were considered as definite (microbiologically confirmed), and probable or possible, when the infection was not microbiologically confirmed, according to the International Sepsis Forum definitions (ISF) for pneumonia, bloodstream infections (including infective endocarditis), intravascular catheter-related sepsis, intra-abdominal infections, urosepsis, and surgical wound infections [53]. When culture results were pending, categorization was reconsidered when the culture results were available. Infections were also registered as community-acquired, ICU-acquired, hospital non-ICU-acquired, or long-term care facility-acquired according to their location of onset.

The CRF allowed the collection of up to 3 concurrent infections per patient present on the study day. According to culture results, infections were categorized as MDRO or non-MDRO. MDROs included carbapenem-resistant *A. baumannii* (CRAB), difficult-to-treat *P. aeruginosa*, CPE, organisms with an extended-spectrum β-lactamase-producing phenotype (ESBL), vancomycin-resistant enterococci (VRE), and methicillin-resistant *S. aureus* (MRSA) (Table S1). If available, further diagnostic methods were performed to classify genetic mechanisms of resistance, including *Klebsiella pneumoniae* carbapenemase-producing *Enterobacterales* (KPC), metallo-beta-lactamases (MBL), oxacillinases (OXA), ESBL-producing organisms, and AmpC beta-lactamases. Non-MDRO of interest included *S. pneumoniae*, *S. pyogenes*, *S. aureus*, *E. coli*, coagulase-negative *Staphylococcus*, *Proteus* sp., *Klebsiella*-*Enterobacter*-*Serratia*-*Citrobacter* (KESC) group, *C. difficile*, among others.

Antibiotic utilization data were also collected and were classified as empiric or targeted, according to the absence or presence of positive cultures, respectively. Antibiotic treatment was further classified as: adequate or inadequate, according to antimicrobial susceptibility testing results; as novel vs. “old”, according to the European guidelines for treatment of infections caused by multidrug-resistant Gram-negative bacilli [54]; and finally, as Access, Watch or Reserve groups, according to the AWaRe WHO classification. [55].

At 60 days post-enrollment, information on mortality status (ICU discharge vs. death) was requested from local investigators. Pending cultures or any other missing data were also collected.

Hospitals were classified as general or specialized and as public or private. We recorded the total number of hospital and ICU beds, the presence of committees for the prevention and control of infection, and antimicrobial stewardship programs. Additionally, data on the frequency of MDRO surveillance for infections and colonization were collected. Methods used to detect types of bacterial resistance were recorded as phenotypic, molecular, or immunochromatographic.

The funder of the study had no role in study design, data collection, data analysis, data interpretation, or writing of the report.

The study is registered with ClinicalTrials.gov, NCT06574776.

### 4.1. Outcomes

The main study outcome was to estimate the prevalence of ICU MDRO and non-MDRO infections. Secondary outcomes included: the prevalence of CPE colonization, ICU mortality, and ICU length of stay (LoS_ICU_). Data were censored for 60 days.

### 4.2. Statistical Analysis

The prevalence of infections was calculated as the number of patients with infections in the participating ICU divided by the number of patients in the ICU on the day of the study; the prevalence of MDRO infections was determined as the number of patients with MDRO infections divided by the same denominator. Variables are expressed as absolute numbers and percentages or medians and interquartile [IQR] ranges. We evaluated differences among patients with and without infections and patients with infections classified as MDRO and non-MDRO. Differences were analyzed using the χ^2^ test or Fisher’s exact test for categorical variables, and the *t*-test or Wilcoxon rank-sum test for continuous variables, as appropriate. All reported *p*-values are two-sided, with a *p*-value < 0.05 considered statistically significant. Missing data were not imputed. We planned to perform a logistic regression analysis to determine independent predictors of ICU mortality and MDRO infections. Independent variables in the bivariate analysis with a *p*-value of <0.20 were entered into the multivariable regression model.

## 5. Conclusions

In this study, we found a high overall prevalence of infections, particularly healthcare-associated infections, and a low prevalence of MDRO infections. CPE colonization acquired during the ICU stay was more frequent in patients with MDRO infections. We did not find an association between MDRO infections and mortality. However, MDRO infections were associated with a longer duration of stay in the ICU and the hospital. These findings highlight the growing challenge of healthcare-associated infections and antimicrobial resistance in pediatric critical care and underscore the importance of robust infection prevention and antimicrobial stewardship programs.

## Figures and Tables

**Table 1:** Characteristics of participating centers.

Characteristics	Centers (*n* = 50)
Hospital category	
Public	37 (73.6%)
Private *	13 (26.4%)
Type of hospital	
General (admission of adult and pediatric patients)	33 (66%)
Specialized (admission of pediatric patients only)	17 (34%)
Number of hospital beds	153 [90–130]
Number of ICU beds	8 [6–15]
Number of patients included per hospital	3 [2–8]
Other resources	
Hospital-acquired infection control committee	48 (96.3%)
Antimicrobial stewardship program	24 (47.2%)
Frequency of CPE colonization screening	
Weekly	40 (79%)
At ICU admission	30 (60%)
Prior to surgery	12 (23%)
Methods used to detect mechanisms of bacterial resistance	
Phenotypic	45 (89%)
Molecular	20 (40%)
Immunochromatographic	34 (68%)

* Private hospitals also include community hospitals and hospitals belonging to the health insurance sector. Data are presented as counts (%), or median [0.25–0.75] percentiles. Abbreviations: ICU: intensive care unit; CPE: carbapenemase-producing *Enterobacterales*.

**Table 2:** Epidemiological characteristics of the study population and comparison between patients with and without infection (*n* = 304).

Variable	All Patients *n* = 304	Patients Without Infection *n* = 167	Patients with Infection *n* = 137	*p* Value
Age (months)	27 [5–88]	25 [5–85]	34 [6–88]	0.44
Gender (male)	168 (55.2)	90 (53.9)	78 (56.9)	0.60
Comorbidity	99 (32.5)	45 (26.9)	54 (39.4)	0.021
Respiratory disease	62 (20.4)	34 (20.3)	28 (20.4)	0.73
Prematurity	52 (17.1)	33 (19.8)	19 (13.9)	0.04
Bronchopulmonary dysplasia	39 (12.8)	29 (17.4)	10 (7.3)	0.009
Congenital heart disease	32 (10.5)	23 (13.8)	9 (6.6)	0.04
Immunosuppression	18 (5.9)	10 (6.0)	8 (5.8)	1.00
Oncologic/Hematologic disease	18 (5.9)	8 (4.8)	10 (7.3)	0.36
Chemotherapy in the previous 6 months	10 (3.3)	3 (1.8)	7 (5.1)	0.12
Obesity	14 (4.6)	7 (4.2)	7 (5.1)	0.71
Chronic liver disease	6 (1.9)	3 (1.8)	3 (2.2)	1.00
Chronic renal disease	4 (1.3)	3 (1.8)	1 (0.7)	0.63
Solid organ transplantation	3 (1.0)	2 (1.2)	1 (0.7)	1.00
Cystic fibrosis	3 (1.0)	1 (0.6)	2 (1.5)	0.59
Diabetes	2 (0.7)	1 (0.6)	1 (0.7)	1.00
Bone marrow transplantation	1 (0.3)	1 (0.6)	0 (0.0)	1.00
**Predisposing factors for MDRO infections**				
Hospital admission in the previous 6 months	141 (46.3)	85 (51.0)	56 (40.9)	0.08
Use of antibiotics in the previous 6 months	149 (49.0)	92 (56.0)	57 (41.6)	0.02
Colonization with MDRO in the previous 6 months	35 (11.5)	21 (12.6)	14 (10.2)	0.52
Time from ICU admission to the study day (days)	7 [2–34]	10 [2–45]	6 [3–17]	0.07
Time from hospital admission to the study day (days)	11 [3–29]	15 [4–50]	7 [3–18]	0.02
Colonization with CPE *	40 (13.2)	21 (12.6)	19 (13.9)	0.87
Time from diagnosis of colonization to the study day (days)	21 [8–77]	24 [14–102]	16 [1–64]	0.30
Mode of infection acquisition:				-
Community	66 (21.7)		66 (48.2)	
ICU	52 (17.1)		52 (38.0)	
Hospital non-ICU	10 (3.2)		10 (5.2)	
Nursing home	9 (2.9)		9 (6.7)	
Healthcare-associated infections **	69 (22.6)		69 (50.4)	-
**Status at admission**				
SOFA at admission	3 [2–6]	3 [1–6]	4 [2–7]	0.03
SOFA on the study day	2 [1–4]	1 [0.3] [8–77]	3 [1.5]	0.0000
**Type of admission**				0.021
medical	232 (76.6)	118 (71.1)	114 (83.2)	
elective surgery	56 (18.5)	40 (24)	16 (11.7)	
emergency surgery	15 (5)	8 (4.8)	7 (5.1)	
Trauma	2 (3.0)	3 (1.8)	6 (4.4)	0.19
**Clinical status on the study day**				
Septic shock	12 (3.9)		12 (8.8)	-
Sepsis	45 (14.8)		45 (32.9)	
**Infection status category**				
Isolation of non-MDRO	54 (17.7)		54 (39.4)	-
Isolation of MDRO	14 (4.6)		14 (10.2)	-
No bacteriologic confirmation (probable or possible infection)	69 (22.6)		69 (50.4)	-
**Outcomes**				
Mortality ***	16 (5.3 [3.0–8.4])	7 (4.2 [1.7–8.5])	9 (6.6 [3.1–12.1])	0.36
Length of ICU stay (days)	22 [8–67]	27 [7–81]	20 [9–64]	0.05
Length of hospital stay (days)	30 [14–87]	32 [11–93]	30 [17–73]	0.02

* Colonization with carbapenemase-producing *Enterobacterales* refers to the detection of the carriage during the incident admission, but prior to the day of the study. ** Healthcare-associated infections represent the sum of infections acquired in the ICU, in hospital settings other than the ICU, and in nursing homes. *** Mortality is expressed as a number, percentage, and CI95% for the percentages. Abbreviations: MDRO: multidrug-resistant microorganisms; ICU: Intensive Care Unit; SOFA: Sepsis-related Organ Failure Assessment.

**Table 3:** Characteristics of patients with infections caused by MDRO and non-MDRO.

Variable	Patients with Non-MDRO Infections *n* = 54	Patients with MDRO Infections *n* = 14	*p* Value
Age (months)	26 [4–93]	43 [9,10]	0.40
Gender (male)	35 (64.8)	9 (64.3)	0.97
Comorbid condition			
Respiratory disease	8 (14.8)	2 (14.3)	0.96
Obesity	2 (3.7)	1 (7.1)	0.51
Diabetes	0 (0.0)	0 (0.0)	1.00
Chronic liver disease	1 (1.9)	1 (7.1)	0.37
Chronic renal disease	0 (0.0)	1 (7.1)	0.21
Immunosuppression	3 (5.6)	2 (14.3)	0.27
Bone marrow transplantation	1 (1.9)	0 (0.0)	1.00
Solid organ transplantation	0 (0.0)	1 (7.1)	0.21
Oncologic/Hematologic disease	5 (9.3)	1 (7.1)	0.64
Chemotherapy in the previous 6 months	2 (3.7)	1 (7.1)	0.68
Human immunodeficiency virus infection	0 (0.0)	0 (0.0)	1.00
Bronchopulmonary dysplasia	3 (5.6)	2 (14.3)	0.27
Cystic fibrosis	1 (1.9)	0 (0.0)	0.79
Congenital heart disease	6 (11.1)	1 (7.1)	1.0
Prematurity	12 (22.2)	1 (7.1)	0.28
Predisposing factors for MDRO infections			
Hospital admission in the previous 6 months	29 (53.7)	5 (31.7)	0.37
Use of antibiotics in the previous 6 months	29 (53.7)	8 (57.1)	0.81
Colonization with MDRO in the previous 6 months	7 (13.0)	5 (35.7)	0.047
Colonization with carbapenemase-producing *Enterobacterales* during ICU stay *	9 (16.7)	8 (66.7)	0.002
Time from colonization to the study day (days)	45 [13–111]	16 [1–42]	0.23
Time from ICU admission to the study day (days)	5 [3–14]	35 [10–78]	0.06
Time from hospital admission to the study day (days)	12 [5–46]	54 [15–107]	0.008
Time from hospital admission to diagnosis of infection (days)	8 [0–30]	73 [14–99]	0.003
Time from diagnosis of infection to the study day (days)	5 [3–12]	8 [2–12]	0.77
Mode of acquisition of infection: Community/ICU/hospital non-ICU/nursing home	17/26/4/4 (33.3/51.0/7.8/7.8)	2/11/0/1 (14.3/78.6/0/7.1)	0.268
Healthcare-associated infection **	34 (63)	12 (85.7)	0.165
**Status at admission**			
SOFA at admission	4 [2–8]	5 [3–9]	0.42
**Type of admission** (medical/elective surgery/emergency surgery)	43/8/3 (80.0/15.0/5.0)	10/3/1 (71.4/21.4/7.1)	0.80
Trauma	3 (5.6)	1 (7.1)	0.82
**Clinical status on the study day**			
Septic shock	6 (11.1)	2 (14.3)	0.67
Sepsis	15 (27.8)	7 (50.0)	0.36
SOFA at the study day	3 [1–5]	3 [3–8]	0.20
**Outcomes**			
Mortality ***	4 (7.4 [2.0–17.9])	1 (7.1 [0.3–33.9])	0.97
Length of ICU stay (days)	25 [12–27]	81 [22–150]	0.0007
Length of Hospital stay (days)	33 [19–83]	116 [46–168]	0.0001
**Antimicrobial use**			
By therapeutic approach			0.874
Empiric	16 (30.0)	4 (28.6)	
Targeted	36 (66.7)	10 (71.4)	
By concordance with antimicrobial susceptibility			0.916
Adequate	46 (85.2)	13 (92.9)	
Inadequate	4 (7.5)	1 (7.1)	
By type of antimicrobial			0.007
New	1 (1.85)	3 (21.4)	
Traditional	50 (92.6)	11 (78.6)	
By AWARE classification (WHO)			0.048
Access	26 (48.1)	5 (35.7)	
Watch	23 (42.6)	5 (35.7)	
Reserve	3 (5.5)	4 (28.6)	

* Colonization with carbapenemase-producing *Enterobacterales* refers to the detection of the carriage during the current ICU stay but prior to the day of the study. ** Healthcare-associated infections represent the sum of infections acquired in the ICU, in hospital settings other than the ICU, and in nursing homes. *** Mortality is expressed as number, percentage, and CI95% for the percentages. Abbreviations: MDRO: multidrug-resistant microorganisms; ICU: Intensive Care Unit; SOFA: Sepsis-related Organ Failure Assessment.

**Table 4:** Characteristics of MDRO and non-MDRO infections.

	Multidrug-Resistant Microorganisms (*n* = 14)							Non-Multidrug-Resistant Microorganisms (*n* = 54)									
Site of Infection	Total Number	*A.∙baumanii*	DT-*P.∙aeruginosa*	CPE	ESBL	VRE	MRSA	Total Number	*S.∙pneumoniae*	*P.∙aeruginosa*	*S.∙aureus*	*E.∙coli*	*S.∙coagulase negative*	*Proteus* sp.	*KESC*	*S.∙pyogenes*	Other
Community-acquired pneumonia	2 (14.2%)						2	17 (23.6%)	4	1	1					3	8
Hospital-acquired pneumonia	-							3 (4%)				1	1				1
Ventilator-associated pneumonia	6 (42.9%)		1	2	3			17 (23.6%)		6	3				1		7
Catheter-related bloodstream infection	3 (21.4%)				3			8 (11.1%)			1		2		2		3
Primary bacteremia	-							1 (1%)									1
*Clostridium difficile* diarrhea	-							3 (4%)				1				2	
Cardiovascular infections	-							-									
Gynecological and obstetric								-									
UTI-community	-							2 (3%)					1				1
UTI-related to catheter	1 (7.1%)			1				6 (8.3%)				3			2		1
Post-surgical meningitis	-							3 (4%)		2			1				
Surgical site infection	1 (7.1%)			1				3(4%)		1	2						
Intraabdominal infection	1 (7.1%)				1			3 (4%)							1		2
Skin and soft tissue	-							1 (1%)			1						
Osteoarticular infection	-							1(1%)			1						
Other	-							4 (6%)					1	1	1		1
Total number	14 (100%)	0	1	4	7	0	2	72 (100%)		10	9	5	6	1	7	5	25

## Data Availability

The data that support the findings of this study are available in Supplementary Materials.

## Supplementary Materials

The following supporting information can be downloaded at: https://www.mdpi.com/article/10.3390/antibiotics14050493/s1, Table S1: List of participating hospitals. Table S2: MDRO included in the study.
