# Supplementary material for: Prevalence and Associated Mortality of Infections by Multidrug-Resistant Organisms in Pediatric Intensive Care Units in Argentina (PREV-AR-P)
Source: Antibiotics (Basel). 2025 May 11;14(5):493. doi: 10.3390/antibiotics14050493 (PMC12108436; doi:10.3390/antibiotics14050493)
Supplement: Supplementary file 1 [file antibiotics-14-00493-s001.zip › antibiotics-3580639-supplementary.pdf]

Supplementary Materials

Table S1: List of participating hospitals (n=50)

Center	Province	Research team	Number of patients included
Hospital Interzonal de Agudos Esp. en Pediatría "Sor Maria Ludovica"	BA	Alancay Alejandra, Torre Valeria, Pablo Castellani, Franco D'Agostino, Agustín Dalceggio<br>Ana Nina Varela Baino	18
Hospital Interzonal General de Agudos "Dr. José Penna"	BA	Rosana Gadea, Diana Portilla	1
Hospital Materno Infantil Dr. Carlos Gianantonio	BA	Roberto Marcelo Pena, Santiago Ayala Torales, Sebastián Cruz, Alejandra Gaiano	2
Hospital Pediátrico Falcón	BA	Macarena Uranga, Leonardo de Lillo, Celia Guantay	4
Hospital "Profesor Doctor Ramón Exeni"	BA	Valeria Grilli, Fabiana Aprile, Matías Penazzi	3
Nueva Clinica del Niño de la Plata	BA	Alicia Vázquez, Mónica Moyano, Juan Alconada	19
Hospital Zonal General de Agudos "Dr. Alberto Eurnekian"	BA	Carolina Osuna, Marta Chamorro	1
Hospital de Alta Complejidad en Red El Cruce "Néstor Kirchner"	BA	Lidia Espinola, Mariana Langard, Marisabel Comas, Karina Cinquegrani, Edgar Amundarain, Sandra Lambert	9
Hospital Interzonal General de Agudos Eva Perón	BA	Hernan Sagasta, Débora Greco, Carolina Salazar, Laura de Aguilar	2
Hospital Escardó de TIgre	BA	Gladys Ferrucci, Patricia Crotti, Diego Lafosse, Carolina Laffont	4
Hospital Interzonal General de Agudos Evita	BA	Patricia Santillán, Rodrigo Corral, Agustina Artillo, Edson González Aguilar, Wanda Re	2
Hospital Interzonal General de Agudos "Dr. Abraham Piñeyro"	BA	Micaela Tolosa, Mario Cuitiño, Aldana	1

		Bustamante, Soledad Martiren, Antonela Culaciati	
Hospital Interzonal Gral. de Agudos "San José"	BA	Josefina Mauro, Candela Luengo	1
Hospital Italiano de San Justo	BA	Laura Ducatenzeiler, Carolina Aquije Obando, Verónica Fernández, José Benso, María Marcó del Pont	4
Hospital Municipal Dr. Raúl F. Larcade	BA	Alejandro Ferioli, Débora Greco, Enrique Serio, María Mastruzzo, Silvia Villa	1
Hospital Municipal de Agudos Dr. Leonidas Lucero	BA	Diego Marcelo Maurizi, Violeta Torres, Marisol Valle, Ivana Tonetto, Lorenzo Olivero	2
Hospital Nacional Alejandro Posadas	BA	Diego Laplumé, Adriana Fernández Lausi, Fernanda Benedetti, Verónica Freire	15
Hospital Interzonal General de Agudos Petrona V de Cordero	BA	María Agustina Casaretto, Johanna Juddith Pérez, Maria de las Mercedes Nano, Susana Guido	3
Hospital Universitario Austral	BA	María de los Ángeles Cuello, Carlos Steer, Pablo Pratesi, Wanda Cornistein, Andrea Novau, Macarena Uranga	9
Sanatorio Trinidad Ramos Mejia	BA	Cecilia Pereyra, Fabiana Garcia	6
Hospital de Pediatría Prof Dr Juan P. Garrahan	CABA	Guadalupe Pérez, Rosa Bologna, Carlos Cambaceres Solana Pellegrini, Graciela Fiorilli, Vanesa Reijtman	33
Hospital General de Niños Pedro de Elizalde	CABA	María Belén Corona, Cecilia Echave	10
Hospital de Niños RIcardo Gutierrez	CABA	María Soledad Areso, Julieta Caridi, Shirly Magee Bahl, Débora Turina, Analía Costas	22
FLENI	CABA	Florencia Escarra, Alejandro Hlavnika, Gustavo Ostales, Andrea Mora, Julieta Camporro	8

Hospital Durand	CABA	Analía Fernández, Celina Hajdinjak, Eugenia Occhiuzzi	3
Hospital Italiano Central	CABA	Marisa del Luján Sánchez, Astrid Smud, Corina Nemirovsky, Mariana de Paz, Vanina Stanek, Noelia Mañez, Facundo Arguello, Emilio Felipe Huaier, Nicolás Lasserre, Luz Guadalupe Ibañez, Karen Cabezas, Carolina Guerra, Florencia Indo, Yolleth Andrade, Emilse Díaz Lobo, Diana Hongn, Yessica Molina, Analía de Cristófano, Victoria Pérez Campione	17
IADT	CABA	Mariana Espina Peña, Sebastián García, Martin Baratelli, Lorena Bracamonte	2
ITAC	CABA	Natalia Pujato, Adriana Marquez, Santiago Galián	2
Sanatorio Anchorena	CABA	Melisa Tatta, Laura Inda, Violeta Cardenas, Carla Sanchez, Claudia Salgueira	2
Sanatorio Franchin	CABA	Cecilia del Valle Barrios, Soledad Areso, Yeseña Cedenio	12
Sanatorio de Los Arcos	CABA	Verónica Paz, Alejandro Gómez, Rodrigo Alzola, Franco Garibaldi	3
Sanatorio Mater Dei	CABA	Cecilia Vera Ocampo, María Inés Sormani, Diego Torres, Soledad González, Gonzalo Echavarría	6
Hospital de Niños de Catamarca	Catamarca	Patricia Carrizo Herrera, Walter Marzola	5
Hospital Andrés Isola	Chubut	Julieta Levite, Emilse Oliva	1
Hospital Regional de Comodoro Rivadavia	Chubut	Soledad Mussini	3
Hospital de Niños de la Santísima Trinidad	Córdoba	Andrés Gomila, Yohana Quiroga Valdez	17
Hospital Nuestra Señora de la Misericordia	Córdoba	Flavio Lipari, Ruth Brito, Mariana Montamat, Sofia Nelli, Melisa Zucotti, Natalia Spitale	2

Instituto de Cardiología de Corrientes	Corrientes	Silvina Villamandos, Laura Peña, Mariano Leonardo Braccini	3
Hospital Materno Infantil Jujuy	Jujuy	Rubén Antonio Camaño	8
Hospital Pediátrico A. Fleming	Mendoza	Margarita Tavella, María Agustina Racioppi, Célica Irrazabal	3
Hospital F. Barreyro de Posadas	Misiones	Verónica Deschutter, Silvia Villordo, Martín Lafuente	7
Hospital Francisco López Lima de General Roca	Rïo Negro	María Laura Ulzurrun, Daniela Durany	1
Hospital Zonal de Bariloche Ramón Carrillo	Rïo Negro	Antonella Espíndola, Carola Curunao, Lucrecia Diaz, Antonella Medoni, Sabrina de Bunder	2
Hospital Público Materno Infantil	Salta	Pamela Celeste Arancibia, Gloria Méndez, Adriana Falco, Beatriz López	8
Hospital San Luis	San Luis	Pablo Vattimo, Nelson Salinas	5
Hospital Regional J.D.Perón Villa Mercedes	San Luis	Alejandra Sofía Cuello, Daniel Elias	1
Hospital de Niños Víctor J Vilela	Santa Fe	Mariela Formaggio, Gabriela Ensink	5
Hospital Italiano Rosario	Santa Fe	María Florencia Galati, Paula Gabriela Urbinati, Daniel Saez, Victoria Emeli Rucci	2
Hospital del Nino Jesús	Tucumán	Paola Deolinda Juarez, Guillermo Lain Fagalde, Mercedes Beatriz Galeano	12
Hospital de Clínicas Pte Nicolás Avellaneda	Tucumán	Luz María Olivo, Noelia Galvez, Luisa Fernanda Arriola, Patricia Chacana, Noelia Jorge	24

Table S2: MDRO included in the study.

MDRO	DEFINITION
Extended spectrum β-lactamase producing microorganisms (ESBL)	Isolation resistant to third and fourth generation cephalosporins; indicates resistance to aminopenicillins, ureidopenicillins, carboxypenicillins, first and second generation cephalosporins (excluding cephamycins), and monobactams. The presence of the enzyme in isolates can be confirmed by phenotypic methods: disk diffusion and automated systems (Phoenix, VITEK) or genotypic methods: PCR for identifying Extended-Spectrum Beta-Lactamase
Carbapenemase-producing *enterobacterales* (CPE),	Carbapenem-resistant isolates in which the presence of the carbapenemase enzyme was confirmed, according to the available diagnostic methods.<br>Genotypic methods: detection of different genes: KPC, OXA, NDM, VIM, IMP.<br>Phenotypic methods: disk diffusion methods and synergy evaluation, immunochromatographic methods, colorimetric methods, and microbiological methods
Difficult-to treat *P. aeruginosa* (DTR-PAE)	DTR is defined as *P. aeruginosa* exhibiting non-susceptibility to all of the following: piperacillin-tazobactam, ceftazidime, cefepime, aztreonam, meropenem, imipenem-cilastatin, ciprofloxacin, and levofloxacin.
Carbapenem-resistant *A.baumannii* (CRAB)	Carbapenem-resistant *Acinetobacter spp* isolates (*Acinetobacter baumannii/calcoaceticus complex, Acinetobacter baumannii*, and other *Acinetobacter* species).<br>94.7% of carbapenem resistance in CRAB (carbapenem-resistant *Acinetobacter baumannii*) is due to OXA-23 oxacillinase, 4.6% to NDM, and 0.7% to OXA + NDM.*
Vancomycin-resistant enterococci (VRE)	*Enterococcus faecium* and *Enterococcus faecalis*, that have developed resistance to the antibiotic vancomycin mainly through the alteration of the target site for vancomycin.
Methicillin-resistant *S. aureus* (MRSA)	*S. aureus* with resistance to all beta-lactams and their combination with beta-lactamase inhibitors (except ceftaroline and ceftobiprole) can be detected using genotypic methods: PCR (detection of the MecA gene) or phenotypic methods: immunochromatographic, according to oxacillin interpretation.

*WHONET Report, Argentina 2023: http://antimicrobianos.com.ar/2025/04/ano-2024/
